# Supplementary material for: Changes in methodological study characteristics in psychology between 2010-2021
Source: PLoS One. 2023 May 10;18(5):e0283353. doi: 10.1371/journal.pone.0283353 (PMC10171647; doi:10.1371/journal.pone.0283353)
Supplement: Attachment — Submitted filename: Response to Reviewers.pdf [file pone.0283353.s001.pdf]

Universität Hamburg
DER FORSCHUNG | DER LEHRE | DER BILDUNG

FAKULTÄT
FÜR PSYCHOLOGIE UND
BEWEGUNGSWISSENSCHAFT

UHH • Fakultät PB • Von-Melle-Park 5 • 20146 Hamburg

To
The Editor and the Reviewers of
PloS ONE

**Dipl.-Psych. Ingmar Böschen**
Insitute of Psychology
Research Methods and Statistics
Von-Melle-Park 5
22761, Hamburg
Germany
Tel. +49 40 42838-5345
ingmar.böschen@uni-hamburg.de
www.uni-hamburg.de

25.8.2022

Response to the reviewers' comments on:

**Changes in methodological study characteristics in psychology between 2010-2021**

Dear editor, dear reviewers,

First of all, I would like to thank you for the opportunity to revise my article and for the helpful comments. I hereby send you the revised version of my manuscript and refer to the proposed changes.

- I have updated the Funding statement as follows:
„This research was financed by a doctoral grant awarded by the Department of Research Methods and Statistics, Institute of Psychology, University Hamburg, Germany. The funders had no role in study design, data collection and analysis, decision to publish, or preparation of the manuscript.“

- The affilliation and address line have been adapted to PloS One style.
Note: Our department has recently been denominated. The name has been updated.

- I submitted a protocol to protocols.io and created a workspace with the data and script to reproduce the analysis: dx.doi.org/10.17504/protocols.io.j8nlk-k775l5r/v1

Thanks for yours and the reviewers time, effort and the helpful comments. I am looking forward to your response.

With kindest regards,

Ingmar Böschen

**Reply to Reviewer 1**
a) Low power = small samples = high uncertainty
Statistical power is undoubtedly as positive property of a statistically analyzed study. As the author mentioned, however, so is noisy measurements. Also, power is linked to sample size and expected effect sizes, and it should be estimated before running a study. Of all these important points, the authors gather data only on frequency of a priori power analysis and sample size.
It would be interesting to see whether study with a priori power analysis showed greater power than study without an a priori power analysis (given the observed effect sizes) or at least whether study with an a priori power analysis employed a larger sample as compared with studies that do not run a power analysis. This would help to really understand the consequences of adherence to “best practices”. We all assume that best practices are beneficial for the quality of research, but nobody brings evidence that they are. A study like this one is an opportunity to check the effects of these practices.

- Thanks for the comment. I agree with you that this correlation is interesting, but I think that this question needs its own manually curated study, since the application of power analyzes is quite heterogeneous (a priori/post hoc, targetted effect size, statistical method), which cannot be distinguished well here.

Coherently, I was expecting the author to gather information about reporting standardized effect size indices. It would be interesting to show effect size increased over time, and if there is a negative correlation with the sample size, as the use of power analysis would imply.

- Thanks for the helpful comment. I have added an analysis regarding the reporting of standard effect measures pre/post 2016 (line 440).

No mention of the level of measurements reliability in the analyzed paper can be found in the results section, even thought the author mentions this property as an important quality of research.

- Thanks for the comment. I have added information about the accurary of the allgorithms in the method section (line 306).

b) The undirected nil-null test scenario
The author claims that directed tests (one-sided tests) improve the quality of researching. Under some circumstances, such as in registered report, many would agree with this claim. However ,what happens in practice is that researchers willing to use one-sided tests are often accused by reviewers to capitalize on their chances to get something significant, because one sided tests are more powerful, and thus more likely to yield a significant result than two-sided tests. So, the lack of one-sided tests (5% before 2015 and 3% after) may simply reflect the more stringent adherence of reviewers to the so-called “best practices”, which usually entails asking for more stringent tests.

- Thanks for the comment. I agree with you that post hoc decisions for one-tailed testing increases the false positive rate and should be labeled bad practice. I pointed this out explicitly in the discussion (line 556). Still, I believe, that one-sided tests are severely underrepresented and that undirected testing should be replaced with equivalence or two one-sided tests against minimal effects of interest. A good overview of this type of testing can be found here:
https://journals.sagepub.com/doi/10.1177/2515245918770963

The other claim in this section regards the pledge to report confidence intervals. Confidence intervals, when computed based on large sample theory, contain exactly the same information than the frequentist inferential tests they come from. They just portrait the information in a different format. Furthermore, they are even more misinterpreted that the corresponding inferential tests. Thus, reporting confidence intervals does not add or subtract anything to the quality of the research, and possibly introduce misinterpretation. Interpreting correctly the intervals, however, can be a positive addition to an article. If I recall well, Cumming's "new statistics" was not about blindly reporting the confidence intervals, but it was about interpreting them and draw conclusions from their width. If one only uses them to check if zero is inside, a t-test/p-value would do the job just fine.

- Thanks for the comment. I specified that (line 153 + 528).

Do the author have evidence that articles that report confidence intervals also interpret them correctly, or they use them in any way which improves the evaluation of the results? Just showing that confidence intervals are reported more often now than in the past, may simply reflect changes in journals policy, without any practical effect on research quality.

- Unfortunately, no statement can be made in this regard as the interpretation of CIs is not focussed here.

c) Questionable research practices
This issue is mentioned in the introduction, but no data are reported to say something about it. This issue may be removed from the introduction, or some creative data may be found to check on its evolution over time. Number of registered reports may be analyzed, or comparisons between registered and not registered studies can be carried out on some key properties. Replications of the same experiment within the same papers will be also interesting, but I guess that this information is more difficult to extract from the data.

- Thanks for the comment. An analysis of the evolution of preregistered studies, has been added in a new result section (line 440).

d) Multiple testing
The statement saying that "[..] the α level or p-values should always be adjusted/corrected for multiple tests" is too general and likely untrue. Assume I have a 3 balanced groups (A,B,C) experiment, and I registered that my theory will be supported if, and only if, A !=C and (A+C)/2!=B. If both comparisons are false, the probability of supporting my hypothesis is alpha^2, which is obviously less than alpha. If only one comparison is "true" (say the second), then my probability of supporting my hypothesis is alpha*(1-beta2), which is always less than alpha, since 0 Adjustment for multiple comparisons is required when one is willing to accept any statistically significant results as valid and supporting a hypothesis.

- Thanks for the comment. I changed the statement according the adjustment of α level from „always“ to „usually“ (line 218).

So, the author should check if adjustment is more likely when no hypothesis is laid out in advance, because that is when adjustment is requirement. Showing that adjustment has increased in frequency may just be a sign that researchers are reducing their a priori hypotheses and increased their HARKing.

- Thanks for the comment. Since no information is extractable on whether hypotheses were laid out in advance, this question remains unanswered.

2) Has the statistical analysis been performed appropriately and rigorously? NO
If I had the option, I would have marked this “partly”. The methods are good and fine, but I’d like to mention the necessity to add control variables to the analyses. The most striking example is the analysis of the number of p-values reported by each paper. Number of p-values may be highly correlated with number of studies reported in a paper and methods used (experimental vs correlational studies).

- Thank you for this helpful comment. I now report the number of p-values per study instead of per article.

Thus, any increase or decrease over time of the number of reported p-values per article may be uncorrelated with the inferential strategies of the researchers, but simply due to a change in format of the journals. In non-randomized designs, furthermore, number of p-values can also be correlated with the use of control variables, as a way to identify possible spurious effects. It would be ironic if more controlled studies were judged as less accurate because they present more p-values than less controlled studies.

- Thanks for the comment. I agree with you on this.

As regards sample size (which is gathered for studying reporting it), it will make more sense to control for effect size. A small study on a large effect size may be as powerful as a large study on a small effect size. In general, sample size is correlated with the generality of the effects one is studying. Surveying detailed opinions about a product may require a much larger sample to obtain stable results than a study counting how many hemispheres there are in the human brain. As mentioned above, it would be also interesting to correlate the sample size with the presence of a priori power analysis.

- As stated above, such an analysis should be examined more closely in a separate study.

I would suggest to elaborate more the theoretical justification and the implications of the different indicators analyzed in the paper, and find more interesting relationships among indicators that could give a more precise and insightful overview of the evolution of the field.

**Reply to Reviewer 2**

1. In the introduction, the author fails to cite the literature militating against significance testing. The author should cite that literature or explain why it is not relevant.

- Thanks for the comment. The literature has been cited (line 164).

2. The issue of power analysis seems problematic too in that if one eschews significance testing, then why perform a power analysis to determine the sample size needed to have a good chance of getting a significant result?

- I do not mean to eschew significance testing, rather I am trying to promote justified analysis strategies with specific, non nil-null hypotheses and adequate sample sizes.

3. Why is there no discussion of the fact that, as most people use confidence intervals the way they use significance tests, there is no difference in practical usage? And why is there no discussion of the fact that a confidence interval is severely limited by the fact that the researcher has no idea of the probability that the population parameter is in the interval?

- Thanks for the comment. I have added a statement regarding this justified concern (line 548):

4. The author does not explain why the analyses performed benefit the field.

- Thanks for the comment. I have added a statement in the discussion (line 508).

**Reply to Reviewer 3**

1. The paper has a not very deep appreciation of some of the criticisms of psychological methods and their limits. At times the writing is overly long and at a very basic level for the issues that are studied in the meta-science research that follows.

We can assume that most researchers know the basics of hypothesis testing without needing to see the formula for a t-test, for example, and citations of sources about the controversy can be cited in passing.

- Thank you for the comment. The formula has been removed.

The Introduction needs to focus on the reason why these standards might be changing and the voices calling for change, rather than on basic-level explanations or unsupported claims. In other words, it needs to spend more time being descriptive about why change might be observed, and less being prescriptive and hinting at the author's opinions about the correct course of action.

a. The Ioannidis claim that most published research findings are false is only valid under conditions of testing very low probability hypotheses, ignoring the many non-flashy studies that test well-grounded ideas. If the rate of hypothesis truth was the 10% or so required for this claim to be true, then why would selections of Registered Reports return confirmation rates of 45% or so? This suggests that hypotheses being tested in psychology are about 50% true with some attrition for type II error under high power.
Scheel, A. M., Schijen, M. R., & Lakens, D. (2021). An excess of positive results: Comparing the standard Psychology literature with Registered Reports. Advances in Methods and Practices in Psychological Science, 4(2), 25152459211007467.
see also
Wegener, D. T., Fabrigar, L. R., Pek, J., & Hoisington-Shaw, K. (2022). Evaluating

research in personality and social psychology: Considerations of statistical power and concerns about false findings. Personality and Social Psychology Bulletin, 48(7), 1105-1117.

- Thanks for the helpful comment. This quote is indeed very pessimistic, but is intended to emphasize the problems in psychological research. I have added the statement, that this claim is exaggerated (line 25) and cited the results of Scheel et al. (line 211).

b. The discussion of power assumes that studies can be spoken of objectively as high or low powered when actually it depends critically on the effect size of interest. The assumption of a medium-sized effect as desirable and a small effect as optimum is nowhere justified.

- Thank you for the comment. The text has been revised.

c. The discussion of issues with Bayesian methods (lines 138-147) needs better context as it relates to the content of the research presented. One gets the impression that the author has a few issues with Bayesian methods but this complex topic needs either more or less detail when presented as a statistical practice that may be growing in popularity.

- Thank you for the comment. The text has been revised (line 166+).

d. The John et al. paper is by no means the last word, in fact it was only the first word, in surveys on questionable conduct. Among other things, it failed to distinguish those who used the practice on one occasion from those who did it habitually. This meta-analysis of over a dozen similar studies is more up to date: Fox, N., Honeycutt, N., & Jussim, L. (2022). Better Understanding the Population Size and Stigmatization of Psychologists Using Questionable Research Practices. Meta-Psychology, 6.

- Thanks for this helpful comment. I have revised the section and cited the proposed literature (line 201).

e. The opinion on corrections for multiple testing is prone to misinterpretation without context. Corrections are only appropriate when all the tests in the family are testing the same null hypothesis of complete equivalence, e.g. among the means of a multilevel factor where one is only looking for any differences as a condition of H1. When testing formally separate hypotheses (including different tests in the same paper, separately theoretically meaningful main effects and interactions in an ANOVA) they are not needed.
Rubin, M. (2021). When to adjust alpha during multiple testing: A consideration of disjunction, conjunction, and individual testing. Synthese, 199(3), 10969-11000.
Gelman, A., Hill, J., & Yajima, M. (2012). Why we (usually) don't have to worry about multiple comparisons. Journal of research on educational effectiveness, 5(2), 189-211.

- Thanks for the helpful comment. I revised the section and discussion of results regarding corrections for multiple testing and cited the proposed literature.

2. In the target journals, the author does not regularly acknowledge that there have often been concrete changes in policy in reporting, which is likely to have shaped the changes shown, depending on the enforcement by editors. By loo-

king at author guidelines and editorials it would be possible and desirable to see how much of the change is journal-driven and how much is author-driven. It can also be seen whether journals are keeping authors to their own standards!

- Thanks for the comment. I have specified this in the discussion (line 516).

3. line 294, "many articles report overly many p-values" - by what standard? This seems more of a personal prejudice than a reasoned argument on desirable statistical content. Some journals publish nine-study papers, for example, and a total of 90 tests can easily come out of such a large program. Thus, the raw number of p-values does not seem to be a good metric of usage let alone a cause for alarm.

- Thank you for the helpful comment. I replaced the number of p-values per article by the number of p-values per study to improve the metric.

4. In table 2, the proportion of p-values in each significance band is of much more interest than the raw figures, and I would want to see them tested pre- and post-2015 like the other parameters in table 1.
However, the analysis does not distinguish between p-values that attach to focal claims, and those that are from auxiliary tests, exploratory tests, tests of equivalence (for gender effects, for example, where gender is not the focus of the research), or manipulation checks. I am not necessarily asking for a more rigorous classification, I know it would depend on content and is not machine-readable. But -- it is important to avoid giving the misleading impression that journals are free to publish "nonsignificant findings" when these are mere footnotes to the main significant findings. In that case perhaps the general prevalence of p-values is not that important after all.

- Thanks for the comment. As Table 1 already indicates, there are only minor changes in the pre/post 2016 proprtion of p-values and recomputable p-values that fall in each significance band. A more detailed analysis for each journal and year was omitted.

5. Are the changes before and after 2015 in Table 1 themselves significant (or Bayes different, etc.)? This is a recurring problem, claiming trends without considering that they could be statistical flukes.

- I can follow your concern and added a paragraph in the method section explaining why no p-values nor CIs are reported (line 308).

6. Again regarding multiple corrections, it is not clear whether the kind of research in a field lends itself differentially to multiple corrections, but I think the author's misunderstanding of the need for this feature leads them to believe that for example every test in a 9-study paper should correct for every other test (alpha = .0001?) yet this might be different if split up into three three-study articles, which is clearly absurd. To be accurate, this treatment would need to go beyond the general vague sense that corrections should be applied, and look at the appropriateness - whether the family is indeed multiple tests that share a common null hypothesis of equivalence.

- Thanks for the comment. I revised the section and discussion of results on corrections for multiple Testing.

7. On sample size, the experiment design is a huge factor that is not discussed and can lead to misleading conclusions when comparing journals. Fields such as psychophysiology that use within-designs can achieve smaller sample sizes with no detriment to actual statistical power. However, for temporal comparisons we are probably on better grounds saying sample sizes have gone up or not in a meaningful way.

- Thanks for the comment. I have added a remark on this to the discussion (line 556).

8. It would be interesting to see if one-tailed testing is especially prevalent for p-values that are two-tailed between .06 and .10. While it is correct to apply this method where hypotheses are stated a priori (e.g. pre-registered) and interest in the other direction of effects is formally renounced, my experience is that it is often used cynically and inappropriately to "rescue" a marginally significant result, even when the other tests are two-tailed.

- Thanks for the comment. As no direct association of a single test result and the use of one-sided testing can be made, this interesting pattern will remain unclear. Post hoc decisions for  one-tailed testing are obviously a questionable research practice. To avoid this misinterpretation I added the requirement to always pre-register studies with one-sided tests in the discussion (line 569-574).

9. line 436: " Although the power of a statistical test could simply be increased by one-sided tests, hardly any study uses directed tests" - increasing power is not a good reason to use directed tests; increasing power by changing the alpha criterion should only be done with a strong justification of having a laxer standard for confirmed results. Having a strong directional hypothesis and NO interest in surprising outcomes in the other direction is the main legitimate justification. If you find it goes the other way, d= 1.0, you still have to report a one-tailed test as nonsignificant!

- Thanks for the comment. I have revised the discussion regarding one-sided testing (line 564-568).

10. The Discussion is mostly summative and does not reflect on the factors that could have contributed to the changes that appear to be true (and we are not sure how solid these changes are because, ironically, there are no p-values, confidence intervals or Bayesian tests associated with the claims of change). It is clear that there is a change in some areas but not every field and journal is buying in, while the strongest changes seem to be driven outright by journal policies. A theoretical model where change and field differences are explainable by a) researcher adoption of changing standards in practices b) utility of those practices to research in the specific field and c) concrete journal requirements informed by a and b as well as d) editors choosing to enforce these requirements, would seem to provide the required intellectual framework.

- Thanks for this comment. The discussion has been revised.
