# Supplementary material for: Changes in methodological study characteristics in psychology between 2010-2021
Source: PLoS One. 2023 May 10;18(5):e0283353. doi: 10.1371/journal.pone.0283353 (PMC10171647; doi:10.1371/journal.pone.0283353)
Supplement: Attachment — Submitted filename: Response to Reviewers.pdf [file pone.0283353.s002.pdf]

UHH Universität Hamburg
DER FORSCHUNG | DER LEHRE | DER BILDUNG

FAKULTÄT
FÜR PSYCHOLOGIE UND
BEWEGUNGSWISSENSCHAFT

UHH • Fakultät PB • Von-Melle-Park 5 • 20146 Hamburg

To
The Editor and the Reviewers of
PloS ONE

Dipl.-Psych. Ingmar Böschen
Insitute of Psychology
Research Methods and Statistics
Von-Melle-Park 5
22761, Hamburg
Germany
Tel. +49 40 42838-5345
ingmar.böschen@uni-hamburg.de
www.uni-hamburg.de

22.12.2022

Response to the reviewers' comments on:

Changes in methodological study characteristics in psychology between 2010-2021

Dear editor, dear reviewers,

thank you for the opportunity to revise my revised article and for the comments, which really helped to further improve the manuscript. I hereby send you the revised version of my article and refer to the proposed changes.

I updated the R Skript on https://github.com/ingmarboeschen/JATSdecoderEvaluation/tree/main/Study_ChangesInMethodologyInPsychology to replicate the current analysis.

Thanks for yours and the reviewers time and effort. I am looking forward to your response.

With kindest regards,

Ingmar Böschen

Reply to Reviewer 1

The revision of the manuscript “Changes in methodological study characteristics in psychology between 2010-2021” addressed some of the reviewers concerns, but I still envisage a wide range for improvement.

The fundamental issue with the manuscript that informed my previous review and the other reviewers comments (as far as I understood them) is the strong discrepancy between the aim of the article and the data that are presented. The article aim, even more so in the revised version, is prescriptive, whereas the data are only descriptive. In the introduction and in the discussion of the paper we found several prescriptive statements regarding what should be done as a good practice and what should be avoided.

Let aside that the proposed good practices are still discussed with little context and implications, the main issue is that the data described in the paper do not tell us much about how much psychological scientists are following those practices, and even less about the quality of their statistical machinery. Data describe some overall trends in research parameters, such as sample size, statistical approach, reporting styles. Evaluation of adherence to the good practices and quality of research required a deeper and more sophisticated analysis, as proposed in the first round of reviews, but the authors deem these analyses not doable with the available data.

I am aware that several of the more sophisticated analyses proposed by the reviewers cannot be carried out with the available data, but this limitation cannot be simply dismissed as a technical one. This limitation suggests that the article aim should be changed. The aim cannot be judging the quality of the published research, or its adherence with good practices, but rather describing the overall trends in statistical practice. As it is now, the paper portraits the wrong message than research lines that changed some of the selected parameters over time, such as reporting less p-values or adjusting for multiple comparisons, are improving their quality, when in reality the evidence reported in the paper is indecisive on this matter.

-> Thank you for the comments. I have revised the article to your concern. However, in my article, I want to highlight and question the overly ritualized use of nil-null hypothesis tests as psychology appears to be facing a replication crisis with many false positives, and empirically support many other authors' calls to move away from this practice (undirected test with H0: theta=0 at alpha=.05), to conduct power analyses, report effect sizes and CIs. In addition, I would like to encourage the preregistration of at least confirmatory research plans.

This general issue is reflected in many specific issues in the paper, especially after revision. I highlight the ones arising after the revision:

*) The manuscript mention increasing sample size as a good practice, and this is one of the least debatable statement. The data show positive trends of this pa-

rameter over time. However, in the discussion is now written "The estimated sample sizes have increased within the examined period from a median of 105 in articles published in and before 2015 to 190 in articles published after 2015. Assuming equally large effect sizes of interest and research designs, this is an indication of increased test power. Since the experimental design also influences the power of a test, smaller sample sizes should not strictly be considered as an indication of low power. Compared to between-subject designs, within-subject designs, as often used in Psychophysiology, can achieve a high power with smaller samples." This comment basically says that the observed increase of sample size is not an indicator of more powerful tests, and so is saying that research may have not improved in quality. But then, what does this parameter say in terms of replicability and best practices? What are the implications of this result?

-> Thanks for the comment. I have added an analysis of median sample sizes in articles with and without repeated measure designs (Table 14) and can now clerify on this: "Assuming that the effect sizes of interest have not changed, this is an indication of increased test power." (line 592)

*) One-tail testing is mentioned as a good practice. The authors state "Besides the fact that any good theory implies the direction of an effect, directed testing represents a simple and cost-saving way to increase the power of a statistical test. In order to avoid doubts about retrospective decisions on test sidedness (which surely is a questionable research practice), intentions for directed testing should always be pre-registered." Why should direct testing be always pre-registered, more than two-tail tests? If one has a strong theory, the direction of the effect can be derived from the theory, so it cannot be changed post-hoc. Furthermore, testing a theory-based directional hypothesis with a two-tail test is equivalent to testing it with a one-tail test at alpha/2, so the test is more stringent. Thus, maybe theory-based hypotheses should be pre-registered, independently of the number of tails one is willing to consider. More importantly, what does the data about one-tail testing has to do with the prescription of pre-registration? How do the authors derive this prescription from the available data? If the authors found a large percentage of one-tail test, one could suspect an abuse which could call for pre-registration. The authors found the opposite (very little use), so it is not clear how the prescription arises from the description. Again, one can be in agreement with the prescriptions mentioned in the paper, but it is difficult to see their relationship with the empirical results.

-> Thanks for the comment. If it was standard to test one-tailed, there would be no need to preregister. But within the present two-tailed standard, one-tailed testing may be suspicious to readers, although it is adequate. I have clarified that (line 604).

*) P-value: "Overall, 68% of the recalculated p-values are significant at $\alpha = .05$. In half of the articles, more than two-thirds of the extracted and three-quarters

of the recalculated p-values are below .05. This finding highlights the demand for highly-powered informed null-hypothesis testing with δ not equal to 0, or equivalence tests that reject the presence of a smallest effect size of interest". How do these finding demand for highly powered informed null-hypothesis testing? It is not clear why 68% of significant p-values is an issue that will be solved by testing against δ not equal to 0? How many of this 68% were pre-registered? How many were simple descriptive correlations or preliminary group comparisons not testing any particular hypothesis of interest?

-> Thanks for the comment. I have revised the statement (line 551)

A final note concerns the justification for the lack of inference on the reported parameters. The authors justify the lack of inference by saying "The results are not reported with p-values nor confidence intervals because the sample is a full, not a random sample of psychological research articles by 12 highly reputed journals". This justification is questionable. The authors aim is to present changes in methodological study characteristics in psychology, and to do that they selected a sample of journals, within which they collected the articles. Thus, they have a sample of articles. Their population is the population of articles published in top journals of psychology, which is not limited to the journals examined in the paper. Otherwise, the articles would not be representative of psychological research, and therefore no longer interesting.

-> Thank you for the comment. I can see your point and added 99.9% CIs for the global comparisons in Table 1 and new Table 14, which shows the median estimated sample sizes in articles with and without repeated measures.

Reply to Reviewer 4

While I agree the usefulness of WEIRD research is limited, I don't see a clear connection between having WEIRD samples and the replicability crisis, as replication studies typically tend to suffer from the same WEIRD myopic approach. Can the author further elaborate on how focusing on WEIRD participants might have contributed to the low replication rate observed in OSC? (The link would be obvious if the failure to replicate WEIRD studies was observed in non-WEIRD studies, but I don't think that is the case).

-> Thanks for the comment. I don‘t see a direct connection of the WEIRDness of samples and the low replication rates in the OSC report either. To me, this is just another critical point in psychological research practice, when the aim is to generalize about humans. I have clarified on that (line 240).

Similarly, while I am aware of the many criticisms on p-values, I would like to read how exactly the author thinks that the reliance on p-values in general, or on nil-null hypotheses in particular, is a potential cause of the replication crisis.

-> Thanks for the comment. I don‘t believe that the reliance on p-values is a critical factor as „Valid P-Values Behave Exactly as They Should" (Greenland, 2019).Rather, I think that the pressure to publish 'significant' results in a setting

without thoughts about power encourages the use of questionable research practices, the effect of which is greatest when testing point null hypotheses.

Besides pre-registration, another solution to deal with researcher degrees of freedom (discussed in the QRP section) involves multiverse analyses (Steegen et al., 2016).

-> Thanks for the comment. I have added a brief introduction to multiverse analysis (line 218) and the result of a search task on mentions of multiverse analyses (line 475). They are very rarely applied in practice.

I understand that a fuller description and evaluation of JATSdecoder can be found in another publication, but still, I would like to read a bit more detail in the current manuscript. For example, how is the use of Bayesian methods identified? Is the occurrence of the term “Bayesian” or “Bayes factor” enough? I guess something more clever had been used (because by this procedure, articles that just discuss Bayesian statistics, rather than apply it, or also included), but I would like to get a rough idea of how this was solved.

-> Thanks for the comment. I have added a brief explanation of how the statistical methods are extracted (line 267). I try not to include discussion papers into the analysis by the preselection procedure of empirical research articles.

I am not sure that using the year 2015 (when the OSC report was published) is a valid or useful marker for possible changes in study characteristics. If the OSC was a catalyst for change, its effect will only be visible a couple of years after 2015, given that research and publication is a slow process. And even if, say, 2017 is used as a demarcation line, it will be hard to disentangle the influence of the OSC and the general increasing awareness about replicability issues (of which the OSC itself was an exponent) and changing journal policies. This is acknowledged in the discussion, but using the publication date of OSC as the demarcation line attributes too much causal weight to OSC (and has, per above, the wrong date, even if it would be warranted to attribute that much causal weight to the OSC). More generally, I doubt the usefulness of any pre-201X vs post-201X analyses, for any X. What can we glean from such a dichotomous analysis that we cannot learn from looking at the year-by-year trends?

-> Thanks for the comment. Indeed, the year 2015 is an arbitrary marker. Still, it enables a more global summary on changes over time. I have revised the statement on splitting at the year 2015 and have removed the reference to the OSC report. (line 253 + 342)

Looking at country of origin is a limited proxy to the WEIRDness of the population pool, at best, especially given the rise of worker markets like mturk and prolific. It is perfectly possible (and even likely) that a researcher from a Western university using mturk as a recruitment tool will collect data from, say, Asian participants. Also the other way around is possible (though less likely). I understand this problem is not easy to solve at the scale the current paper looks at, but it might be an issue worth mentioning.

-> Thanks for the comment. I have revised the section on sample characteristics in the discussion (line 592 + 614) but avoided to cite any company.

It is very hard (for me at least) to grasp trends from tables like, Table 5. Maybe

this information is best presented using a figure (with the corresponding tabled in the appendix for the full quantitative details).

-> Thanks for the comment. Graphics that display the result tables are also very messy (too many journals and years and too big differences between journals) and also difficult to read. I prefer to present the tables with exact numbers.

I was somewhat surprised to see that even if journal policies are in place, the recommended or required practices are taken up at a limited scale only. Maybe this observation deserves more discussion. If policies don't seem to work, what other incentives does the author see to accelerate uptake of statistical reforms?

-> Thanks for the comment. I really had to think about this and didn't come up with any good predictions. Apart from the requirement to register the research with a-priori quality criteria other than theta=0 and to justifiy the sample size with considerations of uncertainty, all the ideas I had were too speculative and mostly unrealistic.

typos:
line 245: extend
line 332: 17$

- Thanks for the advice on typos, which have been corrected.
