# Supplementary material for: Changes in methodological study characteristics in psychology between 2010-2021
Source: PLoS One. 2023 May 10;18(5):e0283353. doi: 10.1371/journal.pone.0283353 (PMC10171647; doi:10.1371/journal.pone.0283353)
Supplement: Attachment — Submitted filename: Response to Editor and Reviewers.pdf [file pone.0283353.s003.pdf]

Universität Hamburg
DER FORSCHUNG | DER LEHRE | DER BILDUNG

FAKULTÄT
FÜR PSYCHOLOGIE UND
BEWEGUNGSWISSENSCHAFT

UHH • Fakultät PB • Von-Melle-Park 5 • 20146 Hamburg

To
The Editor and the Reviewers of
PloS ONE

Dipl.-Psych. Ingmar Böschen
Insitute of Psychology
Research Methods and Statistics
Von-Melle-Park 5
22761, Hamburg
Germany
Tel. +49 40 42838-5345
ingmar.böschen@uni-hamburg.de
www.uni-hamburg.de

24.2.2023

Response to the reviewers' comments on:

Changes in methodological study characteristics in psychology between 2010-2021

Dear editor, dear reviewers,

thank you for the opportunity to revise my article again. I hereby send you the revised version of my article and refer to the proposed changes.

I checked the references and could not find any retracted articles. I identified and solved some problems concerning the DOIs:
- The broken DOI of reference 15 has been corrected
- The underscore in DOI of reference 33 still causes problems when being copied
- The unstable DOI of reference 41 has been removed
- I updated reference 48, which recently has been published at Scientific Reports
- The former reference 51 was removed as the software is available on CRAN now

I updated the R Skript to reproduce the analysis on protocols.io: dx.doi.org/10.17504/protocols.io.j8nlkk775l5r/v1 to the latest version.

Thanks for yours and the reviewers time and effort. I am looking forward to your response.

With kindest regards,

Ingmar Böschen

Reply to Reviewer 4

Ultimately, it is up to the author to decide on the framing of his work, but I still find the framing in terms of the replicability crisis confusing. The author acknowledges himself that some of the methodological aspects studies have little to no bearing the said crisis, so there is a possibility for agreement here. My preferred approach would be to discuss the methodological aspects without much fuzz, and in the discussion make the connection to the crisis, for those aspects for which such a connection is valid.

- Thank you for the comment. I prefer to stay with the current structure of the article and have not made any changes in this respect.

Am I correct to assume that a sentence like "The overall rate of articles using Bayesian inferential methods" is false, and should be "The overall rate of articles mentioning Bayesian inferential methods"? I understand non-empirical papers have been filtered out, but still, an empirical paper saying in the discussion that a fruitful future direction would involve Bayesian analyses would be categorized as using Bayesian analyses.

- Thanks for the comment. As only the identified method and result sections are screened to extract the methodological study features, discussion sections containing references to Bayesian analyses should not lead to detections of Bayesian analyses. Nevertheless, I followed your suggestion and toned down the statements in line 447 + 449.
